# Electric Cardioversion vs. Pharmacological with or without Electric Cardioversion for Stable New-Onset Atrial Fibrillation: A Systematic Review and Meta-Analysis

**DOI:** 10.3390/jcm12031165

**Published:** 2023-02-01

**Authors:** Paritosh Prasai, Dhan Bahadur Shrestha, Eltaib Saad, Angkawipa Trongtorsak, Aarya Adhikari, Suman Gaire, Prakash Raj Oli, Jurgen Shtembari, Pabitra Adhikari, Yub Raj Sedhai, Muhammad Sikander Akbar, Islam Y. Elgendy, Ghanshyam Shantha

**Affiliations:** 1 Department of Internal Medicine, Ascension Health St. Francis Hospital, Evanston, IL 60202, USA; 2 Department of Internal Medicine, Mount Sinai Hospital, Chicago, IL 60608, USA; 3 Department of Internal Medicine, Chitwan Medical College, Chitwan 44200, Nepal; 4 Department of Internal Medicine, Province Hospital, Birendranagar 21700, Nepal; 5 Division of Pulmonary Disease and Critical Care Medicine, University of Kentucky College of Medicine, Bowling Green, KY 42101, USA; 6 Department of Internal Medicine, Division of Cardiology, Ascension Health St. Francis Hospital, Evanston, IL 60202, USA; 7 Division of Cardiology, University of Kentucky, Lexington, KY 40506, USA; 8 Department of Internal Medicine, Division of Electrophysiology, Atrium Health, Wake Forest Baptist Health, Medical Center Boulevard, Winston-Salem, NC 27157, USA

**Keywords:** new-onset atrial fibrillation, cardioversion, electrical cardioversion, pharmacological cardioversion

## Abstract

Background: There is no clear consensus on the preference for pharmacological cardioversion (PC) in comparison to electric cardioversion (EC) for hemodynamically stable new-onset atrial fibrillation (NOAF) patients presenting to the emergency department (ED). Methods: A systematic review and meta-analysis was conducted to assess PC (whether being followed by EC or not) vs. EC in achieving cardioversion for hemodynamically stable NOAF patients. PubMed, PubMed Central, Embase, Scopus, and Cochrane databases were searched to include relevant studies until 7 March 2022. The primary outcome was the successful restoration of sinus rhythm, and secondary outcomes included emergency department (ED) revisits with atrial fibrillation (AF), hospital readmission rate, length of hospital stay, and cardioversion-associated adverse events. Results: A total of three randomized controlled trials (RCTs) and one observational study were included. There was no difference in the rates of successful restoration to sinus rhythm (88.66% vs. 85.25%; OR 1.14, 95% CI 0.35–3.71; *n* = 868). There was no statistical difference across the two groups for ED revisits with AF, readmission rates, length of hospital stay, and cardioversion-associated adverse effects, with the exception of hypotension, whose incidence was lower in the EC group (OR 0.11, 95% CI 0.04–0.27: *n* = 727). Conclusion: This meta-analysis suggests that there is no difference in successful restoration of sinus rhythm with either modality among patients with hemodynamically stable NOAF.

## 1. Introduction

Atrial fibrillation (AF) is a supraventricular tachyarrhythmia resulting from uncoordinated atrial electrical activation leading to an ineffective atrial contraction [1]. It is the most common sustained cardiac arrhythmia, with an estimated prevalence among adults between 2 and 4% and with an expected 2.3-fold rise in its risk due to an increase in life expectancy and an intensified search for undiagnosed AF [1]. It is also regarded as the most common arrhythmia encountered in the emergency department [2]. AF can be asymptomatic or symptomatic as palpitations, chest discomfort/pain, dyspnea, and fatigue and can cause hemodynamic instability [1,2]. AF is associated with increased mortality and morbidity due to associated complications such as thromboembolic syndromes, left ventricular (LV) dysfunction/heart failure, and depression, which can become a significant health cost burden [1]. Acute AF refers to those symptomatic, recent-onset atrial fibrillation episodes where cardioversion is a safe option for treatment if the duration of arrhythmia is shorter than 48 h [2]. It is diagnosed with a standard 12-lead electrocardiogram (ECG) showing no discernible repeating P waves and irregular RR intervals [1].

The emergency management of atrial fibrillation has two approaches, namely, either rate control or rhythm control [2]. The former has an integral role in AF management while the latter controls AF symptoms along with improving quality of life [1]. Immediate rhythm control can be achieved with cardioversion. Electrical cardioversion (EC) is the preferred choice in hemodynamically unstable AF patients [1]; however, there are no clearly established treatment guidelines in terms of rhythm control for hemodynamically stable patients with a new onset of atrial fibrillation (NOAF). Some physicians prefer a drug–shock strategy while other physicians prefer shock-only strategy. In one of the previous studies, no significant difference was found between electric vs. chemical followed by an electric cardioversion strategy. However, both approaches were found to be effective in restoring sinus rhythm and reducing the days of hospitalization [2]. 

In comparison to EC, pharmacologic cardioversion (PC) is easier without the requirement of sedation and backup anesthesia but is associated with the downfall of a low success rate of approximately 50% and side effects of antiarrhythmics. EC is found to have a success rate of 80 to 89% with occasional sinus asystole requiring external pacing [3]. EC can be used as a backup following unsuccessful PC resulting in the successful restoration of the sinus rhythm. 

Owing to a lack of guidelines directing the utilization of either EC only or EC preceded by PC for NOAF in hemodynamically stable patients for rhythm control in the emergency department, the authors conducted this systematic review and meta-analysis of the available pertinent studies to assess the efficacy of EC (shock-only strategy) vs. PC followed by EC if required (drug–shock strategy) and their associated outcomes to further guide future research studies.

## 2. Materials and Methods

This study followed the Preferred Reporting Items for Systematic Reviews and Meta-Analysis (PRISMA) guidelines to identify full-length articles written in English [4]. In addition, the study protocol has been registered in the international prospective register of systematic reviews [5] (PROSPERO ID: CRD42022315427) [6].

### 2.1. Criteria for Considering Studies for This Review

#### 2.1.1. Type of Studies 

In this meta-analysis, data were pooled from randomized control trials (RCT) and observational studies comparing the outcome of EC against PC followed by EC in achieving the successful restoration of the sinus rhythm in new-onset hemodynamically stable atrial fibrillation, as well as reporting any of the outcomes of interest that were considered in this review, including emergency department visits, readmission rates, length of hospital stay, and cardioversion-associated adverse effects. 

#### 2.1.2. Type of Participants

Inclusion criteria included patients above 18 years presenting with acute atrial fibrillation (AF) (defined as the onset of symptoms of less than 48 h) or onset within 7 days of arrival and no left atrial thrombus on transoesophageal echocardiography, who were hemodynamically stable at presentation (defined as a systolic blood pressure greater than 90 mmHg and/or diastolic pressure greater than 60 mmHg) and underwent a cardioversion intervention. Patients with chronic/permanent AF with an unknown or unclear duration of symptoms were excluded. Patients with hemodynamic instability (defined as a systolic blood pressure less than 90 mmHg and/or diastolic pressure less than 60 mmHg) and who required immediate cardioversion, those unable to give consent, those with permanent atrial fibrillation, rapid ventricular pre-excitation, acute coronary syndrome, or pulmonary oedema, whose primary presentation was for another condition (e.g., pneumonia, pulmonary embolism, sepsis), and those who converted spontaneously before randomization were ruled out. Patients with AF secondary to electrolyte disturbances, sepsis, or critical illness, fever or hypothermia, and untreated hyperthyroidism were not included. Patients on regular antiarrhythmic drugs and/or those with a high thromboembolic risk (defined as a CHADS-VAS-2 score of 2 or greater) were also not considered in this study.

#### 2.1.3. Types of Interventions

Studies were included if the participants who met inclusion criteria were assigned to two comparison groups, i.e., the intervention group (electrical cardioversion) and the comparison group (pharmacological/chemical cardioversion followed by electrical cardioversion). Non-comparison studies and studies not meeting the inclusion criterion were excluded.

#### 2.1.4. Types of Outcome Measures

Studies that compared the outcome of interest between the two study groups were enrolled in this review. Studies that did not report the desired outcomes or that did not compare the outcomes between the intervention and control arms were excluded.

### 2.2. Outcomes

The primary outcome of interest was the successful restoration of the sinus rhythm. Secondary outcomes included emergency department (ER) revisits with AF, hospital readmission rate, length of the hospital (LOH) stay, and cardioversion-associated adverse events (AEs). Apart from the clinical outcomes, the baseline characteristics of patients on admission, including age, gender, relevant medical co-morbidities, inclusion and exclusion criteria of the particular studies, and details of interventions in the two study groups were also summarized.

### 2.3. Search Methods for Identification of Studies

A comprehensive literature search was conducted in PubMed, PubMed Central, Scopus, Embase, and Cochrane databases to include relevant randomized control trials (RCTs) and observational studies published until 7 March 2022. A search was performed using MeSH terms which included (electrical cardioversion) or (DCCV) or (shock) in All Text AND “atrial fibrillation” in All Text AND (chemical cardioversion) or (pharmacological cardioversion) in All Text. In addition, articles published in non-English languages which had English translations available online were reviewed. Reviews, case reports, and letters were excluded from the database results before full-text screening.

#### Electronic Searches

The detailed search strategy has been attached in Supplementary File S1.

### 2.4. Data Collection and Analysis

Covidence systematic review software was used to screen studies and extract data. Cochrane Review Manager (RevMan) version 5.4 RevMan5.4 (The Cochrane Collaboration, London, UK) was used for the data analysis [7].

### 2.5. Selection of Studies

All studies were first screened independently based on their titles and abstracts by two reviewers using the Covidence systematic review software (Veritas Health Innovation, Melbourne, Australia) [8]. Next, a third independent reviewer resolved the conflicts. Full texts of the selected studies were then further screened using the same method. Any discrepancy was resolved by consensus among all the reviewers. Data were then extracted from all studies selected via the full-text review for qualitative and quantitative analysis. References of the selected retrieved articles were reviewed for additional potentially relevant articles. Finally, all reviewers independently assessed the risk of bias and cross-checked the selected studies. If there were duplicate studies from the same institution/database, the latest study with the largest number of patients was included to avoid duplication.

### 2.6. Data Extraction and Management

Three reviewers independently extracted the data, which were then verified in the presence of a fourth reviewer. The data extracted included the study details, the inclusion and exclusion criteria, demographic and baseline characteristics of the participants, reported intervention and comparison groups, the primary and secondary outcomes of interest, and the comparison of outcomes between the two study groups. Any disagreements were resolved by extensive discussions among the four reviewers. In cases of missing or incongruent data or the need for additional details, the study’s authors requested other data from certain studies’ authors for further clarification. Data were recorded in Covidence and later exported to RevMan version 5.4 for statistical analysis. Outcomes were measured using a fixed or random effect model for dichotomous variables and the mean difference for continuous variables.

### 2.7. Assessment of Risk of Bias in Included Studies

**Figure 1:**
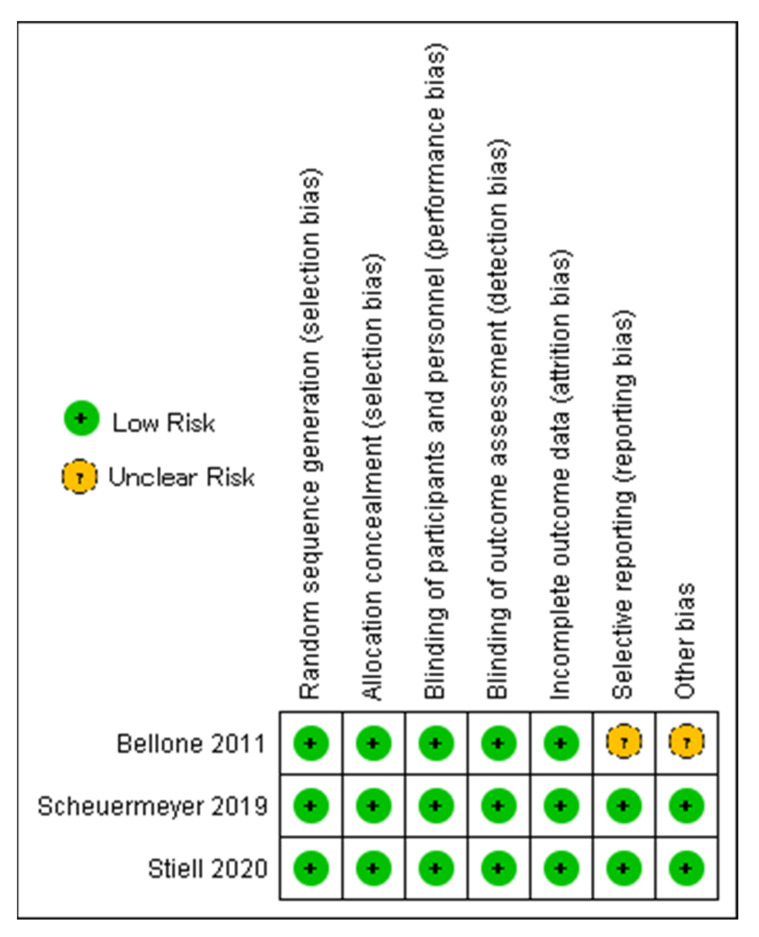
Risk of bias assessment of randomized controlled trials [2,11,12].

Four reviewers independently assessed the methodological quality of the studies using the risk of bias function in Cochrane for RCTs (Cochrane RoB 2.0) [9] and a Risk of Bias Assessment for Non-randomized Studies performed using the JBI tool (Joanna Briggs Institute, Adelaide, South Australia) (Figure 1 and Table 1) [10].

### 2.8. Assessment of Heterogeneity

The heterogeneity in the included studies was determined using the I-square (I^2^) test, using the Cochrane Handbook for Systematic Reviews of Interventions [13].

### 2.9. Assessment of Reporting Biases

Reporting bias was checked using the prefixed reporting of the outcome.

### 2.10. Data Synthesis

Review Manager 5.4 (RevMan 5.4) software of the Cochrane Collaboration (London, UK) was used to perform statistical analysis. In the studies that reported continuous variables as means, mean difference (MD) was calculated. The pooled odds ratios (ORs) and 95% confidence intervals (CIs) were calculated.

### 2.11. Investigation of Heterogeneity

For heterogeneous data, subgroup and/or sensitivity analyses were performed to examine reasons for heterogeneity. Heterogeneity was assessed using the (I^2^) test; the fixed/random effects model was used based on heterogeneity. Publication bias was assessed through the inspection of Forest plots; however, Funnel plots were not used to assess publication bias, as less than ten studies were analyzed.

## 3. Results

### 3.1. Study Characteristics

**Figure 2:**
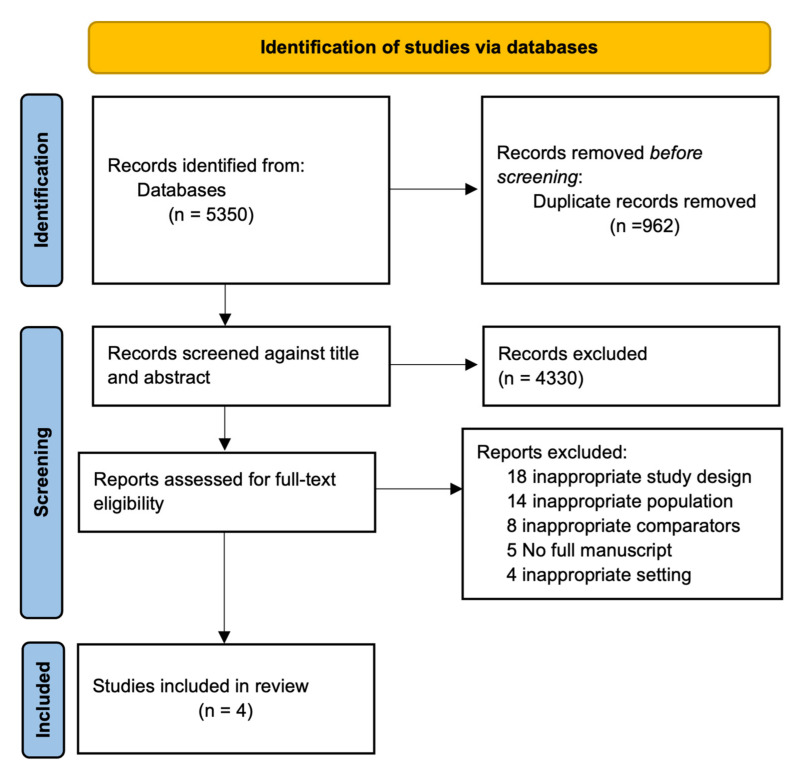
PRISMA 2020 flow diagram for the systematic review.

A total of 5350 studies were identified from the initial database search. After removing duplicates, the 4388 studies underwent a title and abstract review. We then evaluated eligibility, following the inclusion criteria, and only 58 studies were selected for full-text review. Finally, we identified four studies for data extraction based on exclusion criteria. The PRISMA flow diagram for the review is shown in Figure 2.

### 3.2. Qualitative Summary

A total of three randomized control trials and one retrospective observational study were included in the systematic review and meta-analysis. The total sample size (*n*) was 1101 hemodynamically stable patients with acute NOAF and 611 (55.50%) were men. Among the total patients, 441 patients (40.05%) received EC-only/first and 427 patients (38.78%) received PV only/followed by EC. The baseline study characteristics and outcomes have been listed in Table 2 and Table 3.

### 3.3. Quantitative Analysis

#### 3.3.1. Successful Cardioversion

**Figure 3:**
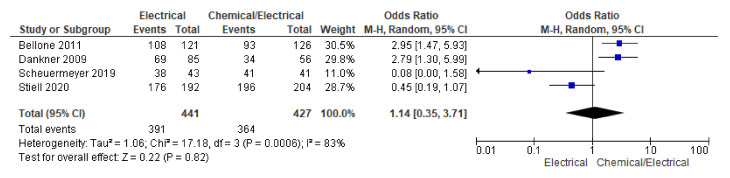
Forest plot showing cardioversion across electrical and chemical followed by electrical cardioversion group using random effect model [2,3,11,12].

Among the four included studies, 88.66% (391/441) successful cardioversion was reported in the electrical group, while 85.25% (364/427) cardioversion was reported in the chemical followed by the electrical cardioversion group. Pooled analysis using the random effect model did not show statistical differences across the two groups (OR 1.14, 95% CI 0.35 to 3.71; *n* = 868; I^2^ = 83%) (Figure 3).

Further analysis which included RCTs only did not show significant differences across the two groups (OR 0.70, 95% CI 0.12 to 4.02; *n* = 727; I^2^ = 86%) (Supplementary File Figure S1).

#### 3.3.2. Emergency Department (ED) Visit

**Figure 4:**
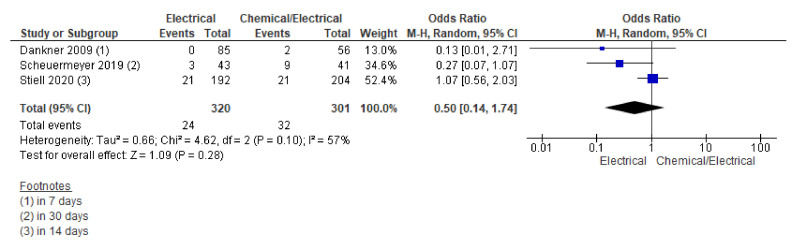
Forest plot showing ED visit rate across electrical and chemical followed by electrical cardioversion group using random effect model [2,3,11].

ED visits were reported in three studies. Pooling data on ED visits using the random effect model showed a trend towards lower odds of ED visits in the electrical arm; however, this effect did not reach the level of statistical significance for the differences across the two groups (OR 0.50, 95% CI 0.14 to 1.74; *n* = 621; I^2^ = 57%) (Figure 4).

Further sensitivity analysis including RCTs only also could not show significant differences across the two groups (OR 0.62, 95% CI 0.16 to 2.34; *n* = 480; I^2^ = 69%) (Supplementary File Figure S2).

#### 3.3.3. Readmission

**Figure 5:**
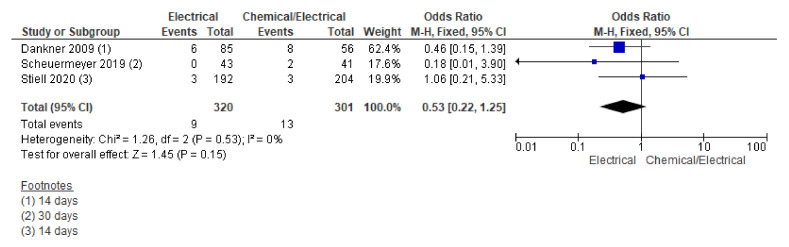
Forest plot showing readmission rate across electrical and chemical followed by electrical cardioversion group using fixed effect model [2,3,11].

Readmission was reported in three studies. Pooling data on the readmission rate using the fixed effect model showed a trend towards lower odds of readmission in the electrical arm; however, this effect did not reach the level of statistical significance for the differences across the two groups (OR 0.53, 95% CI 0.22 to 1.25; *n* = 621; I^2^ = 0%) (Figure 5).

Further sensitivity analysis including RCTs only also could not show significant differences across the two groups (OR 0.65, 95% CI 0.17 to 2.49; *n* = 480; I^2^ = 2%) (Supplementary File Figure S3).

#### 3.3.4. Length of Hospital Stay

Three RCTs reported an overall length of stay in the hospital. A pooling of the data for mean differences using the random effect model from the studies reporting the LOS did not show significant differences across the two groups (MD 0.20, 95% CI −1.42 to 1.82; *n* = 727; I^2^ = 83%) (Supplementary File Figure S4).

#### 3.3.5. Overall, AE

Three RCTs reported overall adverse studies during their study period. A pooling of the data for the adverse events rate using the random effect model did not show statistically significant differences across the two groups; however, a pooled forest plot showed lower odds trending towards the electrical arm (OR 0.25, 95% CI 0.03 to 1.72; *n* = 727; I^2^ = 87%) (Supplementary File Figure S5).

#### 3.3.6. Hypotension, AE

**Figure 6:**
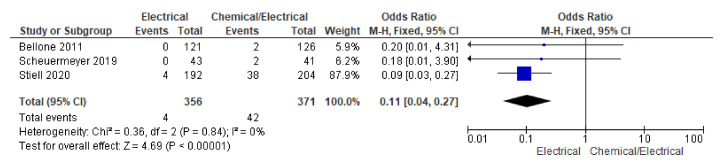
Forest plot showing significant hypotension across electrical and chemical followed by electrical cardioversion group using fixed effect model among RCTs [2,3,11].

Among studies reporting hypotension, pooling data using a fixed effect model showed significantly lower odds of hypotension in the electrical arm (OR 0.11, 95% CI 0.04 to 0.27; *n* = 727; I^2^ = 0%) (Figure 6).

## 4. Discussion

In this meta-analysis including 1101 patients, we assessed the differences between the success rate of cardioversion along with other secondary outcomes between the EC group and the PC followed by the EC group in hemodynamically stable patients with NOAF. More than half of the included patients were males (*n* = 611, 55.5%). Moreover, 40.05% underwent EC while almost one-third of patients (38.78%) received PC followed by EC with no statistically significant difference in the success of restoring normal sinus rhythm between them. The adverse effects were observed in both groups; however, patients who underwent PC followed by EC experienced a statistically significant and higher incidence of hypotension than the EC group, while there were no statistically significant differences in the emergency department (ED) revisits with AF, hospital readmission rate, length of the hospital stay, and overall cardioversion-associated adverse events (AEs) between two groups. The success rate of cardioversion to sinus rhythm was similar between EC and PC followed by EC (88.66% vs. 85.25%). Stiell et al. found a similar success rate of conversion to sinus rhythm between the two groups [2,14]. The studies from Bellone et al., Danker et al., and Scheuermeyer et al. reported a higher success rate for conversion to sinus rhythm in the EC group [3,11,12]. The last study also reported an earlier discharge to home rate in the EC group [11]. On the other hand, Paola et al. reported a similar conversion to sinus rhythm rate between the first attempt EC vs PC group (74% vs. 73%) but a higher success rate in PC followed by the EC group (96% vs. 84%), with the PC group being more cost-effective [15].

Dankner et al. also reported the rate of sinus conversion in the wait and watch approach group (36.4%), which was lesser than in EC-PC group as well as the PC only group (81.2% vs. 60.7%). This is in contrast with the finding of the non-inferiority of the delayed cardioversion with the wait and see approach (69%) compared to early cardioversion (78%) in achieving a sinus rhythm conversion at 4 weeks by Pluymaekers et al. [16].

European Society of Cardiology (ESC) 2020 guidelines for the management of atrial fibrillation outlined the use of either modality for cardioversion (PC or EC) in patients with atrial fibrillation, pointing to the higher success rate of EC modality with no clear preference for either the modality or inconspicuous beneficial significance of EC on the quality of life of patients [1]. A possible explanation for this finding could be the significant differences in sample sizes included in the various studies, as well as the time of the onset of atrial fibrillation to the administration of treatment modality, discrepancies in co-morbidities in patients included in different studies, the use of a variety of modalities in delivering electric current energy and the amount of current used, and discrepancies in the use of different drugs for PC [15]. All these possible hypotheses are a testament to the fact that further studies are required to investigate these factors if we are to clarify these contrasting findings.

In this meta-analysis, there were lower odds of ED visits and readmission among patients treated in EC group than in the PC-EC group. Nevertheless, these odds did not reach a statistical significance level. Stiell et al. reported no significant difference between the two groups regarding ED revisit and readmission (EC vs. PC; 11% vs. 10% and 2% vs. 2%, respectively) [2]. Scheuermeyer et al. reported lower ED revisits among the EC group compared to the PC group (2.4% vs. 12.2%) and no readmission among the EC group (0% vs. 2.4%); however, they were statistically insignificant [11]. Furthermore, there were no significant differences regarding the length of hospital stay between the EC and PC-EC groups. Stiell et al. reported a similar mean total ED length of stay between the EC and PC groups [(7.6 (5.4) vs. 7.1 (5.5) h] [2]. Bellone et al. showed a shorter length of hospital stay in the EC group than in the PC group [(180 (120–900) vs. 420 (120–1400) min] [12].

Our meta-analysis revealed a significantly lower incidence of occurrence of hypotension in the electrical cardioversion group, but no significant differences in the incidence of the occurrence of the overall adverse outcomes between the two groups. Stiell et al. also reported a higher incidence of hypotension in the PC-EC group during the infusion time of one cardiac arrest episode in the EC group and found no difference in the occurrence of overall adverse events between the two groups in their pooled analysis [2]. In the study by Bellone et al., there was an occurrence of atrial flutter, hypotension, cardiac ischemia, and syncope in the PC group (six adverse events); however, hypoxia was reported only in the EC group (one adverse event), and there was no significant difference in the occurrence of the adverse events between them [12]. Scheuermeyer et al. reported a similar rate of occurrence of only minor adverse effects in the two groups with no significant difference [11]. Paola et al. reported a higher occurrence of minor complications such as nausea, vomiting, and observation time comparing two treatment arms in persistent atrial fibrillation [15]. Scheuermeyer et al. reported in another study the occurrence of respiratory compromise due to sedation or electric cardioversion [17]. In our study, the incidence of reporting primary hypotension, benign arrhythmias, agitation, sweating, and apnoea in the EC group was related to sedation during the procedure, while severe complications in the PC group were attributed to the administered antiarrhythmic drugs, and the latter complications were predominately observed among patients with structural heart disease. While we know that the safety profile between the two strategies is comparable with exception of the hypotension, which does not seem to have any clinical consequences, there is an advantage of the drug–shock therapy that should be taken into consideration. Successful conversion with antiarrhythmic agents is more comfortable for the patient and avoids the need for procedural sedation and procedure-related events. Eventually, the choice of the appropriate strategy should come as a shared decision between the patient and the physician [2].

This meta-analysis has some limitations inherent to the nature of the study. Firstly, this is a pooling of data from prior published studies and possible differences in study designs can impact the final findings. In the pharmacological arm among included studies, different antiarrhythmic drugs were used for cardioversion. Those drugs might have a potentially different efficacy and safety profile associated with possible different success rates and adverse effects. Based on the available literature, the ESC guideline recommends vernakalant (excluding patients with recent ACS or severe HF), flecainide, or propafenone (excluding patients with severe structural heart disease) as a drug for pharmacological cardioversion of NOAF [18,19,20,21].

Lastly, paroxysmal atrial fibrillation of a duration less than 48 h has its own tendency to revert back to a normal sinus rhythm. In this case, the efficacy rate of the cardioversion can be falsely high due to the self-resolving nature of PAF < 48 h across both intervention (EC) and control (PC-EC).

## 5. Conclusions

There was no superiority of the electrical cardioversion only method over the pharmacological cardioversion method with or without electrical cardioversion in terms of the success rate of conversion to the sinus rhythm. There was a significantly lower occurrence of hypotension among the EC group; however, this did not affect the length of stay and clinical outcome, which was similar between the two groups. As our result did not show a preference of one strategy over the other, and because it is restricted by the aforementioned limitations, further robust and controlled trials are required to examine the differences between the two arms.

## Figures and Tables

**Table 1:** JBI bias appraisal tool for cohort study.

Checklist	Danker, 2009 [3]
1. Were the two groups similar and recruited from the same population?	Yes
2. Were the exposures measured similarly to assign people to both exposed and unexposed groups?	Unclear
3. Was the exposure measured in a valid and reliable way?	Yes
4. Were confounding factors identified?	Yes
5. Were strategies to deal with confounding factors stated?	Unclear
6. Were the groups/participants free of the outcome at the start of the study (or at the moment of exposure)?	Yes
7. Were the outcomes measured in a valid and reliable way?	Yes
8. Was the follow up time reported and sufficient to be long enough for outcomes to occur?	No
9. Was follow up complete, and if not, were the reasons to loss to follow up described and explored?	Yes
10. Were strategies to address incomplete follow up utilized?	Unclear
11. Was appropriate statistical analysis used?	Yes

**Table 2:** Baseline characteristics of studies.

Author, Year	No. of Patient Population		Age, Years	Gender		CHADS₂ Score			Co-Morbidities			Medications			
				M	F	0	1	≥2	Diabetes	HTN	VHD	AC	AAD	Antiplatelets	Other Cardiac Medications
Stiell 2020 [2]	*n* = 396	Drug–shock (*n* = 204)	60	134	70	65	45	94	1	10	17	66	13	59	101
		Shock-only (*n* = 192)	60.1	126	66	57	54	81	2	14	14	66	13	48	83
Scheuermeyer 2019 [11]	*n* = 86	Chemical-first (*n* = 41)	58	26	15	29	12	0				2	8	18	5
		Electrical-first (*n* = 43)	60	26	17	25	15	3				1	8	19	3
Bellone 2011 [12]	*n* = 247	Propafenone group (*n* = 126)	67 ± 14	65	61				25	65				43	161
		Electrical group (*n* = 121)	68 ± 13	65	56				22	67				50	145
Dankner 2009 [3]		DCC *n* = 85		44	41				6	34	13	19		30	28
		Pharmacological *n* = 56		19	37				6	29	5	15		19	12
		wait and watch *n* = 233		106	127				34	124	23	40		92	69

*n*: number; DCC: direct current cardioversion; M: male; F: female; HTN: hypertension; VHD: valvular heart disease; AC: anti-coagulants; AAD: antiarrhythmic drugs.

**Table 3:** Outcomes of included studies.

Author, Year	Cardioversion Intervention	Cardioversion Control	LoHS Intervention	loHS Control	Re Admission/ Re-Hospitalization Intervention	Re Admission/ Re-Hospitalization Control	Mortality Intervention	Mortality Control	Thromboembolic Events Intervention	Thromboembolic Events Control	AE Intervention	AE Control
Stiell 2020 [2]	Electrical only: 176/192 Chemical: 106/204, 14 days SR: 149/192	Chemical followed by electrical: 196/20, 14 days SR 141/204	7.6 (5.4) h	7.1 (5.5) h	ED visit 14 days: 21/192, Outpatient Visit: 68/192, Hospital admission: 3/192	ED visit first 14 days: 21/204, Outpatient visit: 66/204, Hospital admission: 3/204	0/192	1/204	0	0	Total adverse effects: 5/192 Hypotension: 4/192	Total adverse effects: 53/204 Hypotension: 38/204
Scheuermeyer 2019 [11]	Electrical only: 38/43	Chemical followed by electrical: 41/41	3.5 (2.8–4.8) h	5.1 (3.5–6.3) h	30 days ED revisit: 3/43 Hospital admssion:0/43	30 days ED revisit: 9/41 Hospital admission: 2/41	0	0	Stroke: 0/43	0/41	Total adverse effects: 11/43 Hypotension: 0/43	Total adverse effects: 10/41 Hypotension: 2/41
Bellone 2011 [12]	108/121 patients	93/126 patients	Stay in ED: 180 min (120–900)	Stay in ED:420 min (120–400)	Recurrence of AF during 2 months of follow-up in EC group: 24/91	Recurrence of AF during 2 months of follow-up in PC group: 21/74	0	0	Not reported as secondary outcome	Not reported as secondary outcome	Total adverse effects: 1/121 Hypoten: 0/121	Total adverse effects: 6/126 Hypotension: 2/126
Dankner 2009 [3]	69/85 patients	34/56 patients	Not reported as secondary outcome	Not reported as secondary outcome	ED visit (7 days): 0/85 Hospital readmission (14 days): 6/85	ED visit: 2/56 Hospital admission (14 days): 8/56	0	0	Not reported as secondary outcome	Not reported as secondary outcome		

LoHS: length of hospital stay; AE: adverse events; ED: emergency department; AE: atrial fibrillation; EC: electrical cardioversion; PC: pharmacological cardioversion.

## Data Availability

The data analyzed during the current study are available within the manuscript or in Supplementary Materials.

## Supplementary Materials

The following supporting information can be downloaded at: https://www.mdpi.com/article/10.3390/jcm12031165/s1 Supplementary File S1: Electronic Search Details; Figures S1–S5: Forest plot showing cardioversion across electrical, and chemical followed by electrical cardioversion group using random effect model among RCTs; Forest plot showing ED visit rate across electrical and chemical followed by electrical cardioversion group using random effect model among RCTs; Forest plot showing readmission rate across electrical, and chemical followed by electrical cardioversion group using fixed effect model among RCTs; Forest plot showing the length of stay in the hospital across electrical and chemical followed by electrical cardioversion group using random effect model among RCTs; Forest plot showing overall adverse event rate across electrical and chemical followed by electrical cardioversion group using random effect model among RCTs.
