# Supplementary material for: Electric Cardioversion vs. Pharmacological with or without Electric Cardioversion for Stable New-Onset Atrial Fibrillation: A Systematic Review and Meta-Analysis
Source: J Clin Med. 2023 Feb 1;12(3):1165. doi: 10.3390/jcm12031165 (PMC9918032; doi:10.3390/jcm12031165)
Supplement: Supplementary file 1 [file jcm-12-01165-s001.zip › jcm-2157068-supplementary.pdf]

Supplementary File S1. Electronic search details

Search builder: ((electrical cardioversion) or dccv or shock) AND (atrial fibrillation) AND ((Chemical cardioversion) or (pharmacological cardioversion))

Searched date: 7th March 2022

PubMed

Hits: 893

Link:
https://pubmed.ncbi.nlm.nih.gov/?term=%28%28electrical+cardioversion%29+or+dccv+or+shock%29+AND+%28atrial+fibrillation%29+AND+%28%28Chemical+cardioversion%29+or+%28pharmacological+cardioversion%29%29+

PMC

Hits: 2742

Link:
https://www.ncbi.nlm.nih.gov/pmc/?term=((electrical+cardioversion)+or+dccv+or+shock)+AND+(atrial+fibrillation)+AND+((Chemical+cardioversion)+or+(pharmacological+cardioversion))

Scopus

Hits: 411

Link: https://www.scopus.com/results/results.uri?sort=plf-f&src=s&st1=%28%28electrical+cardioversion%29+or+dccv+or+shock%29+AND+%28atrial+fibrillation%29+AND+%28%28Chemical+cardioversion%29+or+%28pharmacological+cardioversion%29%29&sid=ccb7a69aecf600eeff3ea863afd113d4&sot=b&sdt=b&sl=152&s=TITLE-ABS-KEY%28%28%28electrical+cardioversion%29+or+dccv+or+shock%29+AND+%28atrial+fibrillation%29+AND+%28%28Chemical+cardioversion%29+or+%28pharmacological+cardioversion%29%29%29&origin=searchbasic&editSaveSearch=&yearFrom=Before+1960&yearTo=Present

Embase

Hits: 1124

Link:
https://www.embase.com/?phase=continueToApp#advancedSearch/resultspage/history.4/page.1/25.items/orderby.date/source

Cochrane

Hits: 180

Link: https://www.cochranelibrary.com/advanced-search

Supplementary File S2.

Study or Subgroup	Electrical Events	Electrical Total	Chemical/Electrical Events	Chemical/Electrical Total	Weight	Odds Ratio M-H, Random, 95% CI
Bellone 2011	108	121	93	126	40.9%	2.95 [1.47, 5.93]
Scheuermeyer 2019	38	43	41	41	19.7%	0.08 [0.00, 1.58]
Stiell 2020	176	192	196	204	39.4%	0.45 [0.19, 1.07]
**Total (95% CI)**		**356**		**371**	**100.0%**	**0.70 [0.12, 4.02]**
Total events	322		330			

Heterogeneity: Tau² = 1.83; Chi² = 14.42, df = 2 (P = 0.0007); I² = 86%
Test for overall effect: Z = 0.40 (P = 0.69)

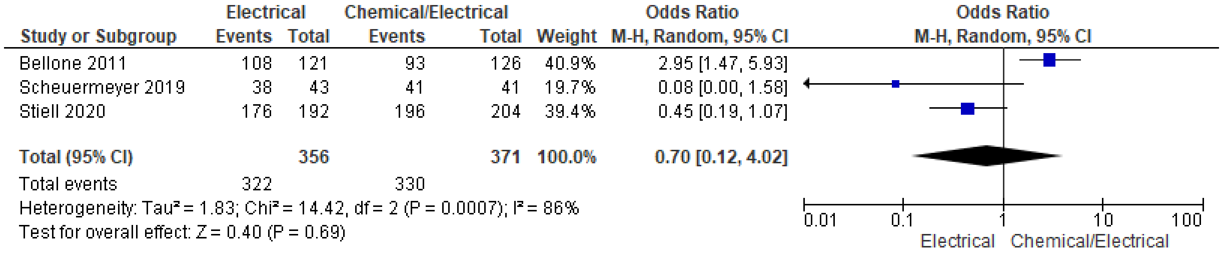

Figure S1. Forest plot showing cardioversion across electrical, and chemical followed by electrical cardioversion group using random effect model among RCTs [2,11,12].

Study or Subgroup	Electrical Events	Electrical Total	Chemical/Electrical Events	Chemical/Electrical Total	Weight	Odds Ratio M-H, Random, 95% CI
Scheuermeyer 2019 (1)	3	43	9	41	39.8%	0.27 [0.07, 1.07]
Stiell 2020 (2)	21	192	21	204	60.2%	1.07 [0.56, 2.03]
**Total (95% CI)**		**235**		**245**	**100.0%**	**0.62 [0.16, 2.34]**
Total events	24		30			

Heterogeneity: Tau² = 0.67; Chi² = 3.19, df = 1 (P = 0.07); I² = 69%
Test for overall effect: Z = 0.71 (P = 0.48)

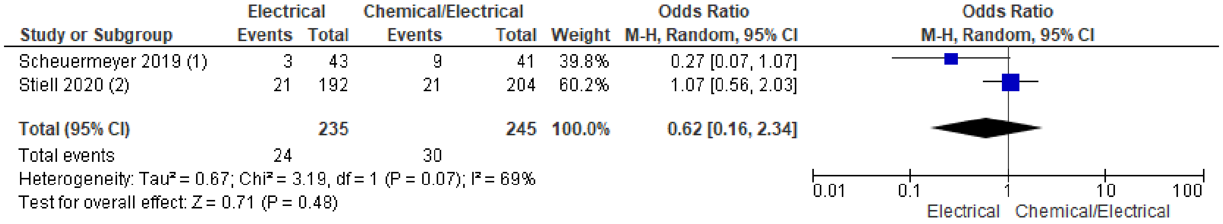

Footnotes
(1) in 30 days
(2) in 14 days

Figure S2. Forest plot showing ED visit rate across electrical and chemical followed by electrical cardioversion group using random effect model among RCTs [2,11].

Study or Subgroup	Electrical Events	Electrical Total	Chemical/Electrical Events	Chemical/Electrical Total	Weight	Odds Ratio M-H, Fixed, 95% CI
Scheuermeyer 2019 (1)	0	43	2	41	46.9%	0.18 [0.01, 3.90]
Stiell 2020 (2)	3	192	3	204	53.1%	1.06 [0.21, 5.33]
**Total (95% CI)**		**235**		**245**	**100.0%**	**0.65 [0.17, 2.49]**
Total events	3		5			

Heterogeneity: Chi² = 1.02, df = 1 (P = 0.31); I² = 2%
Test for overall effect: Z = 0.63 (P = 0.53)

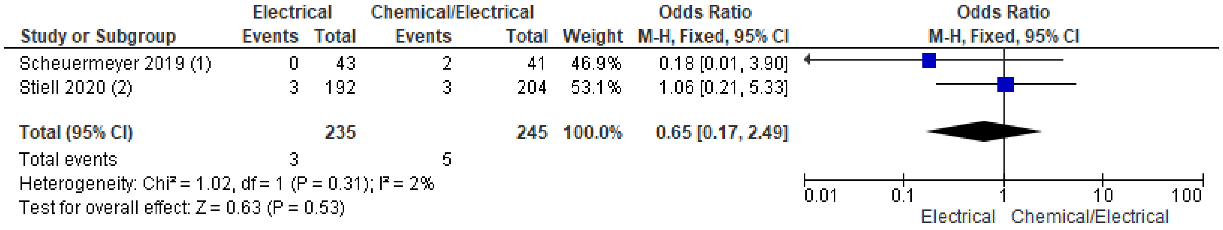

Footnotes
(1) 30 days
(2) 14 days

Figure S3. Forest plot showing readmission rate across electrical, and chemical followed by electrical cardioversion group using fixed effect model among RCTs [2,11].

Study or Subgroup	Electrical Mean	Electrical SD	Electrical Total	Chemical/Electrical Mean	Chemical/Electrical SD	Chemical/Electrical Total	Weight	Mean Difference IV, Random, 95% CI
Bellone 2011	6.86	9.75	121	5.12	3.5	126	27.1%	1.74 [-0.10, 3.58]
Scheuermeyer 2019	3.71	1.53	43	4.9	2.15	41	37.8%	-1.19 [-1.99, -0.39]
Stiell 2020	7.6	5.4	192	7.1	5.5	204	35.2%	0.50 [-0.57, 1.57]
**Total (95% CI)**			**356**			**371**	**100.0%**	**0.20 [-1.42, 1.82]**

Heterogeneity: Tau^2 = 1.64; Chi^2 = 11.57, df = 2 (P = 0.003); I^2 = 83%
Test for overall effect: Z = 0.24 (P = 0.81)

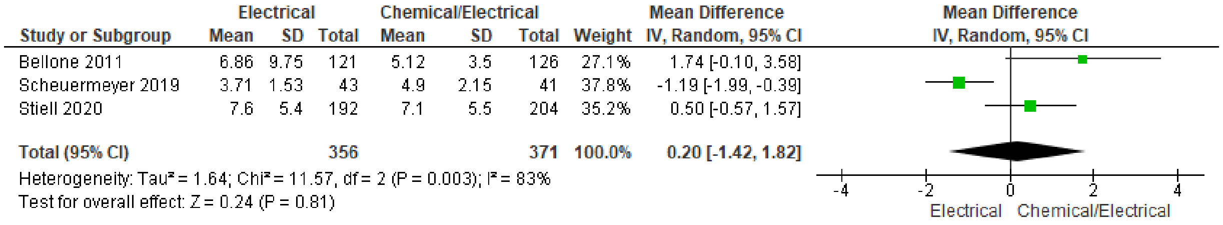

Figure S4. Forest plot showing the length of stay in the hospital across electrical and chemical followed by electrical cardioversion group using random effect model among RCTs [2,11,12].

Study or Subgroup	Electrical Events	Electrical Total	Chemical/Electrical Events	Chemical/Electrical Total	Weight	Odds Ratio M-H, Random, 95% CI
Bellone 2011	1	121	6	126	27.1%	0.17 [0.02, 1.41]
Scheuermeyer 2019	11	43	10	41	36.3%	1.07 [0.40, 2.86]
Stiell 2020	5	192	53	204	36.6%	0.08 [0.03, 0.20]
**Total (95% CI)**		**356**		**371**	**100.0%**	**0.25 [0.03, 1.72]**
Total events	17		69			

Heterogeneity: Tau^2 = 2.47; Chi^2 = 15.00, df = 2 (P = 0.0006); I^2 = 87%
Test for overall effect: Z = 1.41 (P = 0.16)

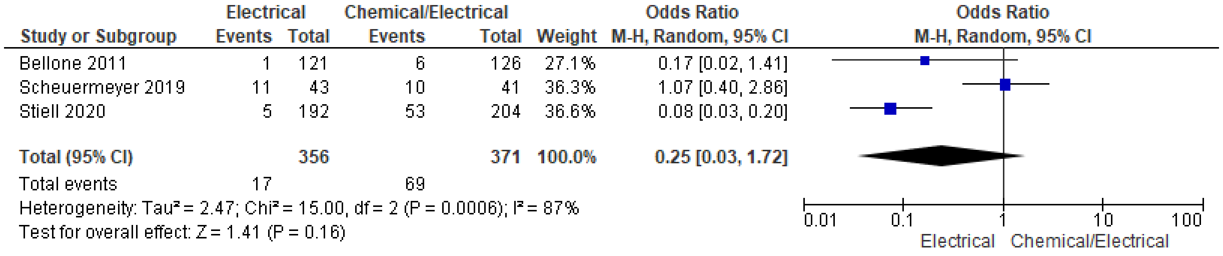

Figure S5. Forest plot showing overall adverse event rate across electrical and chemical followed by electrical cardioversion group using random effect model among RCTs [2,11,12].
